# Cross-sectional household transmission study of *Cryptosporidium* shows that *C. hominis* infections are a key risk factor for spread

**DOI:** 10.1186/s12879-022-07086-y

**Published:** 2022-02-02

**Authors:** Caoimhe McKerr, Rachel M. Chalmers, Kristin Elwin, Heather Ayres, Roberto Vivancos, Sarah J. O’Brien, Robert M. Christley

**Affiliations:** 1 NIHR Health Protection Research Unit in Gastrointestinal Infections, The University of Liverpool, Liverpool, UK; 2 NIHR Health Protection Research Unit in Emerging and Zoonotic Infections, The University of Liverpool, Liverpool, UK; 3 Cryptosporidium Reference Unit, Public Health Wales, Swansea, UK; 4 Field Epidemiology Services, Public Health England, Liverpool, UK; 5 Institute of Infection and Global Health, University of Liverpool, Liverpool, UK; 6 Swansea Medical School, Swansea University, Swansea, UK; 7 Present Address: Public Health Wales, Cardiff, UK

**Keywords:** *Cryptosporidium*, Protozoa, Sporadic disease, Household transmission, Person-to-person, Secondary spread, Zoonoses, Gastrointestinal infection, Risk factors, Epidemiology

## Abstract

**Background:**

Infection with the *Cryptosporidium* parasite causes over 4000 cases of diagnosed illness (cryptosporidiosis) in England and Wales each year. The incidence of sporadic disease has not been sufficiently established, and how frequently this arises from contact with other infected people is not well documented. This project aimed to explore potential transmission in the home and attempt to identify asymptomatic infections, which might play a role in transmission. Risk factors and characteristics associated with spread of infection in the home were described including any differences between *Cryptosporidium* species.

**Methods:**

The study identified cryptosporidiosis cases from North West England and Wales over a year and invited them and their household to take part. Each household was sent a study pack containing study information and a questionnaire, and stool sample kits to provide samples from consenting household members. *Cryptosporidium*-positive stool samples, identified by immunofluorescence microscopy, were characterised using molecular methods to help describe any patterns of transmission. Characteristics of households with and without additional cases were described, and compared using odds ratios (OR) and a multivariable logistic regression identified independent risk factors for household transmission. Data collection ran for one year, beginning in September 2018 with an initial pilot phase.

**Results:**

We enrolled 128 index cases and their households. Additional illness occurred in over a quarter of homes, each reporting an average of two additional cases. The majority of these were undiagnosed and unreported to surveillance. This burden was even greater in households where the index case was infected with *C. hominis* versus *C. parvum*, or the index case was under five years old, with mums and siblings most at risk of secondary infection. Only having an index case of *C. hominis* was independently associated with transmission in the multivariable model (OR 4.46; p = 0.01).

**Conclusions:**

*Cryptosporidium* was a considerable burden in the home. At-risk homes were those where the index was less than five years old and/or infected with *C. hominis*. Of particular risk were female caregivers and siblings. Hygiene advice should be specifically directed here.

This work provides evidence for humans as sources of *C. hominis* infection and that person-person is a key pathway. We recommend that all stools submitted for the investigation of gastrointestinal pathogens are tested for *Cryptosporidium* to better capture cases, inclusion of speciation data in routine surveillance, and the consideration of specific clinical advice on prevention for high-risk homes.

**Supplementary Information:**

The online version contains supplementary material available at 10.1186/s12879-022-07086-y.

## Background

*Cryptosporidium* is a protozoan parasite which can infect humans and other animals, and the most prevalent species identified in humans are *Cryptosporidium parvum* and *Cryptosporidium hominis* [1, 2]. Cryptosporidiosis is the subsequent diarrhoeal disease following infection with *Cryptosporidium*. The disease affects all ages and although generally self-limiting, can be life threatening in some immune-compromised patients. Following an incubation period of between 2 and 10 days acute symptoms can include non-bloody diarrhoea, abdominal cramps, vomiting and/or nausea, low-grade fever, lethargy and general malaise. The parasite has a complex life cycle and characteristics which favour faecal-oral and environmental transmission routes, which may facilitate outbreaks via person-to-person (*C. hominis and C. parvum*) or animal-to-person (*C. parvum*) contact, as well as indirect transmission through ingestion of water and food contaminated with infectious oocysts [3].

Public Health England (PHE) receive laboratory reports of over 4000 diagnosed cases per year (2000–2012 data) in England and Wales, however, research indicates that many infections may go undiagnosed, and the true incidence of disease may be much greater [4, 5]. Risk factors and associated exposures are often hypothesised or identified from outbreak investigations. However, recognised outbreaks may only represent a small proportion of cases [6]. Other routes to infection may be at play in sporadic cases, or indeed in localised outbreaks that are missed by surveillance.

Prior work has examined the contribution of case contact on sporadic disease, highlighting person-to-person spread [7, 8]. Hunter et al. additionally reported that changing children’s nappies was a specific risk factor for infection with *C. hominis* whether the child was symptomatic or not, and a Norwegian study looking at follow-on spread after two outbreaks observed asymptomatic secondary transmission [9]. Other studies have demonstrated an increased risk of illness or infection associated with prior contact with a symptomatic individual [10–12]. Studies of *Giardia*, a similar gastrointestinal parasite, have recently been undertaken in the UK, and supports that for these gastrointestinal parasites, secondary spread and person-to-person transmission seems a likely and under-recognised route of transmission [13, 14].

Existing work does suggest that the home is a particular setting for infection, and that case contact is additionally risky where close contact is more likely [8, 10–12]. This makes biological sense given the faecal-oral route of transmission of oocysts that are already sporulated and infectious, and the higher prevalence of infection in younger children who may require help with toileting [10, 15–17]. This has been further buttressed by large-scale reports of spread in the home following outbreaks [18–20] which may well drive additional, sporadic cases.

We designed an observational study across North West England and Wales [21] to examine additional infections in the home of a laboratory confirmed case of *Cryptosporidium*, in order to describe characteristics associated with transmission and to help inform public health messaging on preventing spread of disease at home.

## Aims

The aims of this study were to estimate how much additional *Cryptosporidium* infection happens in the home where there is a symptomatic, laboratory confirmed case, and to describe characteristics associated with transmission in the home. (We use the term ‘transmission’ to mean any apparent onward spread of disease originating from a case, whilst recognising that disease may have occurred before the identification of our ‘index’, and this may represent secondary or even tertiary levels of spread). Tables 1 and 2 show the case and household definitions.

**Table 1:** Case definitions

Index case	
The first case from a household identified in the surveillance system (person reported to a PHE/PHW surveillance system(s) following detection of *Cryptosporidium* in a faecal sample, with a specimen date in the study year)	
Additional household case	
A person in a household of an index case, with self-reported similar symptoms (in questionnaire) that started within two weeks of the index case’s onset, with or without a *Cryptosporidium* positive stool sample	
Asymptomatic carrier confirmed case	
A person in a household of an index case with: no reports of similar illness (in questionnaire); AND	
A *Cryptosporidium* positive stool sample

**Table 2:** Household definitions

Household	
Two or more people (not necessarily related) living at the same address in North West England or Wales who share cooking facilities and share a living room or sitting room or dining area [29]	
Household member	
A person who normally resides in the household and regularly shares food or toilet facilities (Public Health England, 2017b)	
Household contact	
A household member in a home where an index case has been identified	
Household with transmission	
A household that has at least one additional household case	
Household without transmission	
A household that has one case (the index case)	

### Objectives

- To estimate the amount of additional illness in the home of an index case
- To estimate the prevalence of asymptomatic carriage in households with an index case
- To identify specific household-level and case characteristics associated with homes that have additional cases

## Methods

The study population comprised residents of North West England and of Wales.

The study began recruiting from England in October 2018 and in Wales in Jan 2019 and ran for 12 months in each area.

Assuming that the rate of household transmission, defined as the proportion of households with more than one case, is anywhere between 0 and 20% [13, 20, 22–25], a range of required sample sizes was estimated (between 100 and 402 households). We anticipated recruiting a sample size of 100 households [10% of 1000 cases per year (PHE data, 2015)] based on resource and feasibility.

**Fig. 1:**
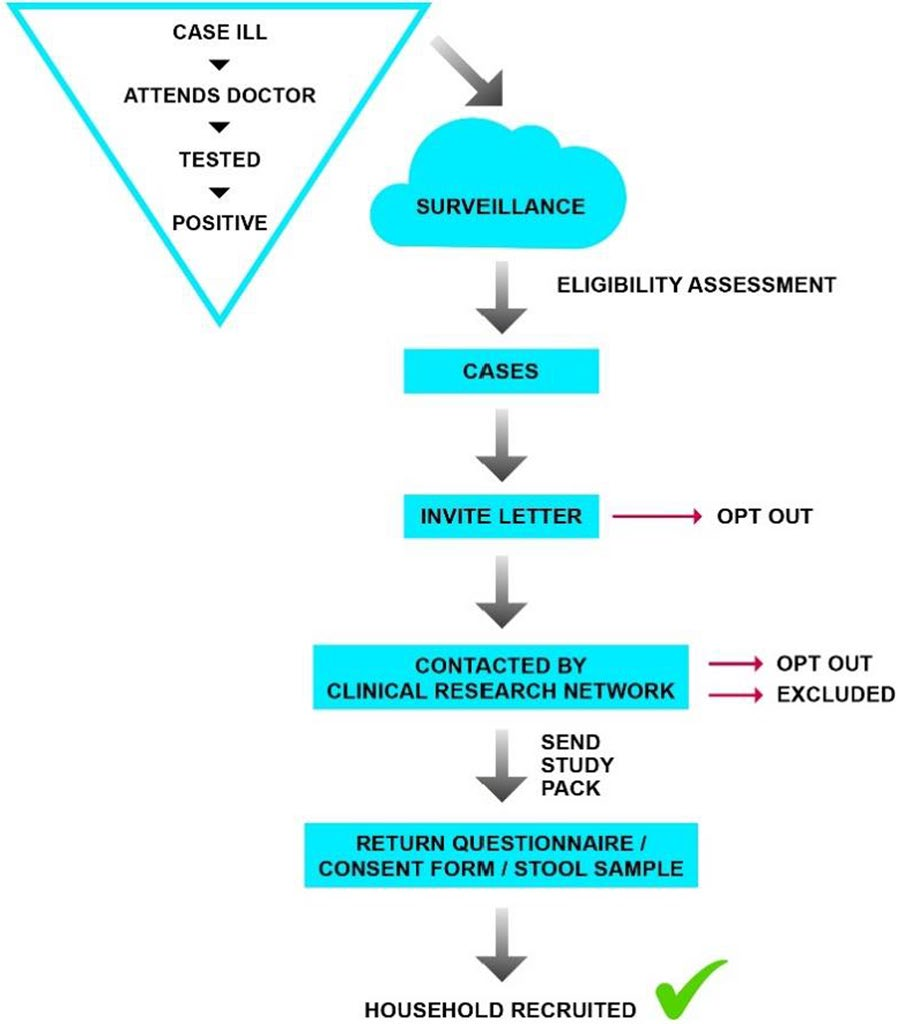
Recruitment flow

Figure 1 outlines the recruitment process. Potentially eligible index cases were laboratory confirmed and reported cases of *Cryptosporidium* identified from the two well-established surveillance systems that capture laboratory notifications in England and Wales. Index cases were excluded if they lived in a single person household or were resident in an institution or shared living. Index cases were sent an invite letter outlining the study, and following that were given a 2-week period in which to opt out.

The contact details of index cases were shared securely (using internally agreed practices) with NHS research nurses at the Clinical Research Network North West Coast (CRN). The research nurses contacted those index cases who did not return an opt-out. Cases were contacted where possible by telephone. Study packs were posted for discussion in the home.

The study pack contained a questionnaire and consent form (one per household) and stool sample (Fe-Col®) collection kits for all participating household members. The index case was not required to supply another sample. A household was considered recruited when they returned any element of the study pack.

The questionnaire (Additional file 1: Questionnaire data) was designed to collect data on the demographic composition of the household, the clinical details of the index case and any other symptomatic household members. Questions also captured household variables, including the number of bedrooms and bathrooms, outside space and animals. We also asked about nappy changing and toilet training in the home, and about general hand-washing behaviour. We asked about activities of the household in the two weeks prior to the index case’s onset, based on known exposures for *Cryptosporidium*, to help elucidate possible co-primary cases. One questionnaire was completed per household.

Diagnostic stools from confirmed cases of *Cryptosporidium* were sent to the Cryptosporidium Reference Unit laboratory for species identification by real-time PCR or sequencing the 18s gene [26] as is usual clinical practice [27].

Samples from household members were submitted by post to the national Cryptosporidium Reference Unit’s laboratory and scored against the Bristol stool scale (BSS). They were then screened only for *Cryptosporidium*, using immunofluorescence microscopy (IFM) (CryptoCel, TCS BioSciences) and an in-house real-time PCR targeting the 18S gene (“CRU18S” assay) [28]. Samples testing negative by both methods were discarded^.^

Samples positive by either IFM or the screening PCR were taken forward to undergo *Cryptosporidium* species identification initially using an in-house, duplex real-time PCR designed to identify *C. parvum* and *C. hominis* [26]. For any screen positive samples that did not amplify with the *C. hominis* or *C. parvum* primers, the 18s amplicons were sequenced to identify any other species (or identify a false-positive screen). *Full laboratory protocol available on request.*

Questionnaire information was entered into MS Access, pseudonymised, and analysed using Stata v12 (StatCorps). Missing data items were excluded individually, but not entire records.

A household with more than one case (of any species) was initially categorised as a household with transmission. We compared household and case characteristics between households with and without additional cases. Additional illness included anyone reporting compatible symptoms within two weeks of the index case and/or a confirmed laboratory case with or without symptoms.

We calculated the following:

- The secondary transmission rate/prevalence within households (number of cases in the home/numbers at risk in the home, number of households with additional cases/number of households);
- The amount of asymptomatic carriage among those exposed to symptomatic cases (number of asymptomatic carrier cases)
- Odds (OR) of additional symptomatic illness by case/household characteristics.

Categorical variables were compared using chi square tests and continuous data using Wilcoxon rank sums, where appropriate. We used backwards stepwise logistic regression to identify independent risk factors for additional illness in the home. All risk factors that had a p value less than 0.2 in the univariate analyses were considered in a multivariable analysis. Age less than five years old and sex were retained in the final model, as they are known to be associated with infection risk. The final model included risk factors that were significantly associated with the occurrence of at least one additional case in the home.

## Results

The study year ran from October 2018 to October 2019 for England, and January 2019-

January 2020 in Wales. Unavoidable, but short-lived, recruitment issues led to a possible dip in enrolment in two periods: January and June 2019.

Over 1000 cases were reported to both surveillance systems over the study year (n = 1030). After the application of the exclusion criteria, 1016 eligible index cases were identified.

Ninety-nine questionnaires were returned, along with 123 household member stool samples.

**Fig. 2:**
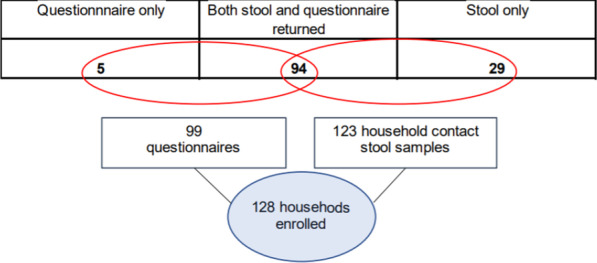
Number of households enrolled by study elements returned

Using either of these elements as consent to enrol, we enrolled 128 index cases and their households into the epiCrypt study in the one-year period of recruitment (response rate 12%) (Fig. 2). Over half of these were resident in the North West of England (n = 76; 59%) and 41% were recruited from Wales (n = 52). This amounted to 413 participants overall, of which 285 were household contacts of an index case.

### Stool samples

We were able to locate the corresponding diagnostic stool samples from 109/128 (85%) index cases. Overall, 259 household member stool samples were returned to the reference laboratory, which, along with the 109 index case samples, yielded a total stool sample count of 368.

Household size ranged between two and seven, with a median of four people per household (mean = 3.4). All participating households were families rather than friends or housemates.

Almost half of households (n = 61; 48%) had at least one child under five living in the home and eight (6%) had an infant under one year old. Eleven (9%) households had at least one person over 65.

Of the 109 index case samples tested, most were *C. parvum* (59.7%; n = 65) followed by *C. hominis* (33.0%; n = 36). Also identified were *C. cuniculus* (n = 3), and *C. ubiquitum* (n = 2). (Table 3).

**Table 3:** Species of index cases

Index case species	All samples	%
*C. parvum*	65	59.7
*C. hominis*	36	33
*C. cuniculus*	3	2.8
*C. ubiquitum*	2	1.8%
Cryptosporidium species unable to be identified	1	0.1
*Cryptosporidium* not detected	2	1.8
Total	109	100

Index case ages ranged from 9 months to 78 years old with a mean age of 22 (median = 12). Females represented 49.6% (n = 63) of index cases, and males 50.4% (n = 64). There was a difference in the distribution of age among the sex categories, with male cases tending to be younger (n = 127; p = 0.030). There was no significant difference in the age distribution among cases of *C. hominis* versus *C. parvum* (p = 0.257).

The index case was a child under five years old in almost 30% of the homes (n = 38; 29.7%) and two-thirds of those were male (n = 25; 65.8%; p = 0.023).

Table 4 shows clinical symptoms reported. Clinical information was only available for those index cases who returned a questionnaire (n = 99). The most frequently reported symptom was diarrhoea (95%) followed by stomach pain (78%). Less than half of cases reported both diarrhoea and vomiting (49%).

**Table 4:** Main symptoms reported by index cases

Symptom	Frequency reported (N = 99)	%
Diarrhoea	94	94.9
Vomiting	49	49.5
Nausea	51	51.5
Pain	77	77.8
Fever	44	44.4
Headache	28	28.3

More than a quarter (n = 27; 27.3%) of cases reported some other symptom(s). These most frequently included foul-smelling stool, sleep disturbances, lethargy and exhaustion, loss of appetite, and joint pain. Among *C. parvum* cases there were more reports of high temperature (57% versus 24% in *C. hominis* cases; p = 0.007).

Length of illness in the index cases ranged from one to 90 days, with a median of 14 days (mean = 18). In 96% of cases (n = 85) symptoms persisted for 7 days or more, and 60% of index cases reported persisting symptoms for at least two weeks (n = 53). There was no relationship between symptoms reported and length of illness. Males were more likely to report a longer illness with a median symptom time of 21 days, versus 15 days among female cases (p = 0.003). *C. hominis* cases were longer than *C. parvum* (20 vs 16 days; p = 0.004).

Additional similar illness in the home within two weeks of the index case was used as a proxy measure for households with transmission.

Twenty-seven of the recruited households (27%) indicated that there had been at least one case of additional illness in the home within two weeks of the index case. Of these, 10 (37%) were prior to the index case.

Fifty percent of all participants reported compatible gastrointestinal illness. The proportion of household contacts that reported symptoms (attack rate) was 31.4%: excluding the index cases, 76 additional cases of compatible illness were reported, out of 242 household contacts. However, 28/81 (34.6%) individuals who did not report symptoms submitted stool samples that were scored a BSS of 6 or 7, indicating diarrhoeic consistency.

The number of cases of additional illness reported (i.e. excluding the index) per home ranged from one to four (n = 25; 2 records excluded due to nonsensical value). On average, 1.8 additional cases were reported per household. This was generally higher in homes where the index was less than five years old. (Table 5).

**Table 5:** Number of additional cases per household, plus range data, by age of index case

Number of additional cases	Index > 5 years old	Index < 5 years old	Total
1	9 (69.3%)	4 (33.3%)	13 (52.0%)
2	3 (23.1)	2 (16.7%)	5 (20.0%)
3	1 (7.7%)	5 (41.7%)	6 (24.0%)
4	0 (–)	1 (8.3%)	1 (4.0%)
Total additional cases	13	12	25
Range	1–3	1–4	
Median	1.0	2.5	
Mean	1.38	2.25	

In 16 (59%) of those homes reporting additional cases the index case was male (p = 0.484) and in 44% (n = 12) the index was less than five years old (p = 0.084). No statistically significant differences were detectable in the length of the index case illness between households that did and did not report other compatible illness (p = 0.838) or by the number of additional cases in the home (Spearman’s rank correlation p = 0.543). On average, 13 days elapsed between onset in the index case and the next case (median = 10 days). The shortest of these was zero days, possibly a co-primary case; these data did not allow for further examination of this.

The number of additional cases reported was greater in households where the index case was infected with *C. hominis* (rank sum p value = 0.03). Less than 20% of the *C. parvum* index cases reported additional illness in their household (19.6%), compared to 48% of the *C. hominis* index cases (p = 0.010).

**Fig. 3:**
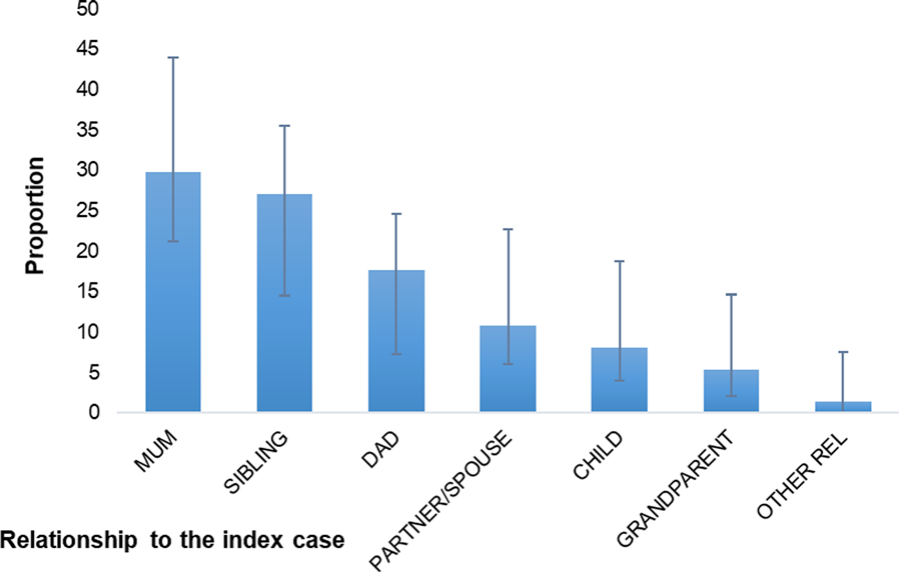
Proportion of additional illness in households by relationship to the index case. *N = 72. Two records excluded though lack of data on these variables

Figure 3 shows the proportion of all additional cases in homes, by the relationship to the index case. The most affected persons in the family* were mothers, who represented 30% of this additional illness burden (n = 22; 95% CI 21.2–43.9). This was followed by siblings, who represented 27% of illness (n = 20; 95% CI 14.52–35.46).

Table 6 shows results from the univariable analysis.

**Table 6:** Households reporting selected exposures and characteristics, by whether or not participants reported other compatible illness within two weeks of the index case, with odds ratios and 95% CI

Characteristic of home and case (exposure)	Households (N = 99) reporting that exposure		Households with additional reported illness (A8_Else = 1, n = 27)		Households without additional reported illness (A8_Else = 0, n = 72)		Odds ratio	95% CI	p value
	n	%	n	%	n	%			
Index case is *C. hominis* ^(vs *C. parvum* (n = 76))^	25	32.9%	12	54.5%	13	24.1%	3.78	1.171, 12.236	0.01
Fewer than 1 toilet per person ^(n = 96, excl. 3 values)^	72	75.0%	23	88.5%	49	70.0%	3.29	0.839, 18.729	0.06
Index case attends nursery	21	21.2%	9	33.3%	12	16.7%	2.50	0.786, 7.651	0.07
Children (5 years old or under) in household	48	48.5%	17	63.0%	31	43.1%	2.25	0.898, 5.728	0.08
Index case under 5 years old	31	31.3%	12	44.4%	19	26.4%	2.23	0.792, 6.158	0.08
Crowded (fewer than 1 bedroom per person) ^(n=97, excl. 2 missing values)^	37	38.1%	13	50.0%	24	33.8%	1.96	0.708, 5.378	0.15
Nappies/potty training anyone in home	32	32.3%	11	40.7%	21	29.1%	1.67	0.591, 4.582	0.27
Pets in household	55	55.6%	17	63.0%	38	52.8%	1.52	0.563, 4.246	0.36
Index case in nappies or toilet training	23	23.2%	7	25.9%	16	22.2%	1.23	0.369, 3.735	0.70
Index shares bed	39	39.4%	11	40.7%	28	38.9%	1.08	0.392, 2.906	0.87
Length of illness > 14 days ^(n = 89, 10 missing values)^	41	46.1%	12	46.2%	29	46.0%	1.00	0.361, 2.767	0.99
Both diarrhoea and vomiting in the index case	48	48.5%	13	48.1%	35	48.6%	0.98	0.398, 2.411	0.99
Length of index case's illness > 7 days ^(n=89, 10 missing values)^	79	88.8%	23	88.5%	56	88.9%	0.96	0.197, 6.242	0.95
Most deprived deciles ^(lowest 5 IMD vs top 5) n=82^	28	34.1%	7	33.3%	21	34.4%	0.95	0.280, 3.011	0.93
Index cooks regularly for home	34	34.3%	9	33.3%	25	34.7%	0.94	0.322, 2.604	0.90
Female index case	46	47.9%	11	40.7%	35	48.6%	0.73	0.266, 1.943	0.48
Total household members ≤ 3	47	47.5%	10	37.0%	37	51.4%	0.56	0.225, 1.379	0.21

The variable most strongly associated with transmission was the infecting species of the index case. Among homes that reported transmission, there was a preponderance of *C. hominis* cases versus *C. parvum* cases and this exposure was three times more likely in homes with additional cases (OR 3.78; p = 0.01).

Homes with additional cases were twice as likely to report an index case less than five years old (OR 2.23; p = 0.08) with 44% of households with transmission reporting this exposure, compared to just over a quarter of homes without additional cases (26.4%). Additionally, this relationship remained when examining any children under five in the home, not just an index case. Having an index case who attended a nursery was more than twice as likely to be reported in homes with transmission (OR 2.5; p = 0.07).

Although not statistically significant, being in a home with three or fewer people was reported in a greater proportion of those homes without additional cases (OR 0.56; p = 0.21). This is supported by the other crowding indicators; less than one toilet per person (OR 3.29; p = 0.06) and less than one bedroom per person (OR 1.96; p = 0.15) were both reported more often in homes where there was transmission.

Table 7 reports results from a logistic regression analysis of those significant variables (p ≥ 0.2) from the univariable analysis.

**Table 7:** Logistic regression model of variables (retaining age < 5 in the model)

Variable (index case characteristics)	Odds ratio	Std. error	z	P >|z|	95% CI	
*C. hominis*	4.46	2.68	2.48	0.013	1.37	14.53
Attends nursery	4.21	5.14	1.18	0.239	0.38	46.14
Less than 5 years old	0.91	1.07	− 0.09	0.931	0.09	9.23
Sex—Female	0.64	0.37	− 0.77	0.44	0.20	1.99
*-cons*		*0.11*	− *2.97*	*0.00*	*0.06*	*0.57*

Associations with transmission in the home were features of the index case: being infected with *C. hominis* versus *C. parvum*, and attending nursery. Crowding indicators and nappy/potty use dropped from the model (likely being features of homes with children). Only having an index case of *C. hominis* was independently associated with transmission in the home in the multivariable model.

Overall, 10/128 (7.8%) households in this study had laboratory confirmed infection in at least one other person in the home. These ten homes with confirmed additional infection yielded 28 household member samples in total. Of these 28 samples, 12 (42.9%) were confirmed positive for *Cryptosporidium* (4%; n = 12/259 household samples). The households comprised one additional household member in each of eight homes, and two household members apiece in the remaining two homes. Table 8 shows a breakdown of this by species of the index case (n = 109). Overall, ten households in this study had confirmed infection in at least one other person (7.8%). Six household members were confirmed in homes with *C. hominis* index cases (17%), three in homes with a *C. parvum* index case (5%), and one (50%) where the index was *C. ubiquitum*. These were all in homes where we were able to identify a corresponding original index case sample. These ten homes with confirmed additional infection yielded 28 household member samples in total. Of these 28 samples, 12 (42.9%) were confirmed: this comprised one household member in each of eight homes, and two household members apiece in the remaining two homes.

**Table 8:** Number of households that had any member samples confirmed as *Cryptosporidium*, by the index case result

Index case species	Total samples	Number that were household member (not index) samples	%
*C. cuniculus*	3	0	0%
*C. hominis*	36	6	17%
*C. parvum*	65	3	5%
*C. ubiquitum*	2	1	50%
*Cryptosporidium* not detected	2	0	0%
*Cryptosporidium* species unable to be identified	1	0	0%
Total	109	10	9%

We were able to confirm infection in two individuals’ samples who reported no symptoms: both were adult males from different households, both *C. hominis*. Both were parents of an index case (of type *C. hominis*). In both cases, other symptomatic illness was reported across the home.

## Conclusions

This exploratory study has highlighted several characteristics of *Cryptosporidium* cases, and of the environment in which they live, that might be correlated with spread of infection. The significant independent factor in multivariable analysis was an index case infected with *C. hominis*.

Our sample recruitment was limited in size, and more households would have increased our precision. However, despite a definite lean towards recruitment of families rather than other household compositions, the characteristics of our included participants were fairly typical of both North West England and Wales. Participants were mostly comprised of 25 to 44-year olds but we also had a decent proportion of young children represented (21% under 5 years), with male cases tending to be younger. This fits with what we know about the descriptive epidemiology of *Cryptosporidium* in England and Wales [30]. The index case was a child under five years old in almost 30% of the recruited households and two-thirds of those were male. This is supported by other examinations of cases in the UK and beyond that demonstrate an increased prevalence in infants and young children [7, 8, 30, 31] but could also relate to larger households giving rise to more opportunity for exposure. The households that took part originated from all socioeconomic areas, but there were slightly more households from the less deprived geographies. This is consistent with the profile of *Cryptosporidium* infection across England and Wales where the most deprived areas appear slightly underrepresented [32]. This might reflect difference in access to, or use of, services, or may be a reflection of differences in recruitment and participation [33, 34].

More than a quarter of index cases reported symptoms other than diarrhoea and vomiting, including nausea, abdominal pain, and headaches. Moreover, vomiting was not frequently reported at all, occurring in less than half of the index cases, which has been noted previously [16, 20, 35] Symptoms differed somewhat by age with nausea, headache, and stomach pain occurring more among older cases and vomiting in the younger cases, particularly males. This could be due to differences in the symptom profile of infecting species. Differences in symptom presentation have been identified before, in an outbreak of *C. hominis*, where headache and abdominal pain were more common in female cases [35]. However, as a high proportion of the cases were children, it is important to remember that we are relying on secondary reports of illness, usually via parents. Self-reported illness can be fraught with reliability issues, especially with non-clinically obvious symptoms like pain or nausea, which may be difficult for a young child to describe or articulate. Nonetheless, vomiting in children as a symptom of *Cryptosporidium* infection has been demonstrated before [8], and may be an important presentation to note; the presence of vomiting does not exclude it from a differential diagnosis in gastrointestinal investigations.

Of additional interest, this study revealed males reported a longer illness as did cases of *C. hominis*. A study of sporadic disease in the UK reported a mean duration of acute symptoms for patients with *C. hominis* that was two days longer than *C. parvum* cases [8]. One interesting finding in this study was that additional, and possibly secondary, cases in the home were not as long-lived as those in the index cases and might point to a decreased virulence in person-to-person spread [36]. This has been evidenced before in homes with transmission of gastrointestinal pathogens, where secondary cases’ average duration of illness was more than half that of primary cases [25].

Persistent and substantial burden of illness on the individual as well as on the home overall is well corroborated in other literature which has revealed duration of symptoms for *Cryptosporidium* infection far beyond IID of other aetiologies [37–40]. Crucially the longevity of illness might also amplify spread, by potentially increasing the length of time the oocysts are shed [36, 41, 42], although here there was no association between length of illness and burden of additional cases in the home. Nonetheless, complications this long lasting and potentially burdensome warrant further examination.

An analysis by species revealed that less than 20% of the *C. parvum* index cases reported additional illness in their household (19.6%), compared to 48% of the *C. hominis* indexes (p = 0.01). This result is in line with similar studies evidencing *C. hominis* as a species particularly associated with people, and probably the person-to-person transmission pathway. A case–control study in the Netherlands [12] found that *C. hominis* cases in particular were three times more likely than controls to have been exposed to a case in the home and were less likely to live in homes with lots of adults. Also, in those years where *C. hominis* was the predominant circulating species, other risk factors such as food items were reported as associated with decreased odds of illness. This work adds to the body of evidence that sources for *C. hominis* infection may be exclusively human and that person-person transmission is the most likely pathway [8, 43].

Almost two additional cases occurred, on average, in households with an index case. Additionally, the analyses suggested that almost a third (31%) of people in the home could be expected to get ill from transmission of infection. This burden was even greater in households where the index case was infected with *C. hominis* or the index case was under five years old. Risk of infection in settings with young children has previously been demonstrated and is known to facilitate spread [8, 20, 44]. This work further buttresses that person-to-person is a specific transmission pathway but is first study to quantify the burden that this exacts on the home.

If most index cases are young children, and mums make up the burden of secondary cases, then it is plausible that the driver here is direct contact, in a caring capacity; undertaking activities which likely put the main carers in the home at high risk. In a study in the UK, Hunter et al. found that changing children’s nappies was a risk factor specific to *C. hominis* [8] and the Netherlands recently reported similar results [12] specifying that *C. hominis* cases were more likely than controls to have been exposed to a case in the home. Additionally, the authors reported corroborating indicators supportive of this exposure, including living in smaller homes, and living with children.

Siblings were also affected considerably by secondary infection, but where the adult was an index case, their children were less frequently the secondary case. It has been documented before that mums and siblings are most at risk of *Cryptosporidium*: in a follow-on study in Norway, with 12 and 13-year-old index cases, a 17% secondary transmission rate was mainly comprised of female caregivers and siblings [20]. This is mirrored among other gastrointestinal aetiologies [25], and indeed for *E. coli* O157 it has been suggested that separation of siblings might be a key intervention in reducing secondary cases [45].

Gender roles are known to influence both patterns of exposure to infectious agents and the treatment of infectious disease [46]. Caring for the sick carries an increased risk of exposure, especially for diseases that are spread directly from person-to-person and in most societies females are more likely to care for the sick than males [47]. The heterogeneity of contact within the home has been examined in respiratory diseases such as Influenza and Pertussis, and studies found that contacts between mother and children and between siblings are most prevalent [48]. Several cost and burden of illness studies have been undertaken in the Netherlands, which have considered the economic and societal impact of gastrointestinal infections. Overall, there is a considerable burden on productivity due to absence from work for the ill or the caregiver(s) [49], and one study estimated that in 15% of cases where a child was ill, a parent had to remain off work [50]. An additional analysis considering the longer-term manifestations of *Cryptosporidium* in particular reported similar burdens on productivity, with additional impact on disability adjusted life years (DALYs) due to recurring diarrhoea and long-term joint pain [51]. Further work confirming and examining this disparity for *Cryptosporidium* would be a welcome addition to work to describe the economic and societal burden of this disease.

This work did not reveal any considerable proportion of asymptomatic infection. *Cryptosporidium* was detected in 12/259 (4%) of household members’ samples of whom two were asymptomatic, giving a prevalence of asymptomatic infection of 2%. From the small number of relevant studies, carriage of *Cryptosporidium* appears to be low at between 0.1 and 1.3% [52, 53] although this has once been demonstrated as high as 9% following an outbreak of *C. parvum* in Norway [20]. Identification of true carriage is difficult as we tend to capture diarrhoeal cases and it is likely that all of the index cases here will have sought clinical assessment following symptoms. In addition, recrudescence of symptoms complicates the identification of differences between true asymptomatic infection and shedding of oocysts in an asymptomatic period. An asymptomatic prevalence of 2% would be in line with carriage expected for the UK [53] but the design of this work did not allow any examination of this contribution to spread.

The time between initial onset of illness in the home and sample retrieval from household contacts was variable, and often long. This does raise some uncertainty about the capability of the tests used to confirm infection. Given that we have already demonstrated differences in length of illness by species, it would not be implausible that asymptomatic, or indeed less protracted secondary infections, might lead to shorter shedding times [25]. If this were the case, the detection power of the tests may be reduced by the time samples are received at the laboratory. Also, a small sample size limits the power to truly detect asymptomatic infections. The true asymptomatic infection burden may well be under ascertained here and previous work reiterates that lack of detection by routine diagnostic methods does not necessarily equate to lack of infection [54, 55]. There are complex biological and social factors that affect surveillance data capture, of which illness severity has been shown to be important [56] and one person’s idea of ‘being ill’ might differ from another’s. Nonetheless, this result is not insignificant. If asymptomatic infections are indeed few, rather than this being something to be dismissed, actually this indicates that if infected you are more than likely to be ill, and we know that with this illness comes considerable symptomatic burden, and the risk of longer term sequelae [16, 57]. As such, this makes tackling preventable secondary transmission of infection a crucial issue of public health importance.

*Cryptosporidium* was detected in 12/259 (4%) of household members’ samples. This is much smaller than expected if the self-reported clinical illness does truly represent secondary infections. An explanation for this may be the lag time from illness to receiving household samples and the likelihood of detecting *Cryptosporidium*. Despite a range of laboratory testing methods, including PCR, the results demonstrated that confirmation was more likely in specimens taken during, or soon after, a case’s symptomatic period. The average time between the index cases' specimen date and the first household member specimen was 43 days. Oocysts might also be shed intermittently but the study design did not allow for repeat sampling.

Additionally, using a clinical indicator of BSS made no difference to microbiological confirmation: all of the diarrhoeic specimens were subsequently unable to be confirmed as infected with *Cryptosporidium*. Conversely, all those household member samples that were confirmed, had formed stools. This supports a prior recommendation to eliminate stool consistency as a testing inclusion criterion in local laboratories in England and Wales *(McKerr & Chalmers, 2020—personal communication)*.

This study detected possible household transmission of *C. ubiquitum*, with an index case and a confirmed household infection. Unfortunately, the questionnaire element of the study was not returned for this home and so further examination of exposures was not possible. But this was an interesting find: it is an unusual species, sources of infections in humans are not entirely clear and transmission between people has never been demonstrated [58].

The variable most strongly associated with additional cases in the home was the infecting species of the index case. The Netherlands recently reported similar results [12], specifying that *C. hominis* cases were more likely than controls to have been exposed to a case in the home. Additionally, the authors reported corroborating indicators supportive of a person-to-person pathway, including living in smaller homes, and living with children. Although not independently associated with transmission in the logistic regression model, this study did highlight similar associated exposures, with homes with additional cases twice as likely to report the index case being a child less than five years old or attending a nursery.

This work continues to buttress the existing literature but highlights quite clearly that differences in species and transmission are quite likely. At risk homes can be identified as those where the index is less than five years old and/or is infected with *C. hominis*. Of particular risk are mums and caregivers, and siblings, and targeted hygiene advice should be specifically directed here.

The demonstration of a *C. hominis*-specific burden provides another argument for swift and complete characterisation of isolates, with results fed into local and national surveillance data. There is certainly a public health, and economic, argument for interventions to reduce not only primary infections with *Cryptosporidium*, but also subsequent spread. This might include work to provide more targeted advice for individual *Cryptosporidium* patients or during outbreaks, and these strategic and population-level approaches are critical given the lack of licensed treatment for this infection in the UK. This evidence does reinforce the importance of speciation and subtyping of isolates where at all possible, in order to better understand the clinical course of disease for the patient or population and administer appropriate interventions and advice.

A study of this kind is not without its limitations. Proving transmission is a difficult task, and studies oftentimes are unable to examine this to ascertain specific differences in cases or identify modifiable risk factors, and very specific study designs are required to examine this. The ubiquitous nature of *Cryptosporidium* and of its exposures make untangling these exposures and demonstrating causality difficult in a study of this set-up. Our sample was small, although participants were largely representative. Nevertheless, this study was intended as an exploratory piece, and the evidence presented suggests that *Cryptosporidium* does transmit readily in the home environment, and that person-to-person could be the transmission pathway.

Additionally, our limited understanding of the background prevalence of asymptomatic infection of *Cryptosporidium*, and its effectors, make it difficult to identify its importance in spread of disease in contained settings, and this study did not reveal a large amount of asymptomatic infection. The study design was not appropriate to demonstrate if an asymptomatic carrier was shedding oocysts or was infectious to others in the home. However, previous work on secondary transmission data has mainly stemmed initially from outbreaks, and data rarely include laboratory confirmation of secondary cases [20]. The epiCrypt study is unique in that it has allowed for an examination of secondary cases at both species level and with further typing. A larger scale study of sporadic infections would continue to build on our understanding of species-specific risks of spread and also could examine heterogeneity in subtype populations [59].

Difficulties arise distinguishing between primary and secondary infections as close contacts often have similar exposures [20] and the clinical course of *Cryptosporidium* infection can result in variable incubation, symptoms, and onset between individuals making verifying person-to-person transmission and differentiating true secondary cases from co-primary challenging. Further genotyping of some samples is ongoing which may support household level investigations of directionality and population mixing. By sequence analysis of the gp60 gene, 31/40 (78%) typable *C. hominis* samples were subtype IbA10G2 which predominates in the UK and much of Europe [31], but our epidemiological findings may not translate to settings where other subtypes are more prevalent. Since this work was completed, there has been a dramatic change in the epidemiology of *Cryptosporidium* in the UK with *C. parvum* becoming the predominant species during the COVID-19 pandemic, following lockdown interventions at the end of March 2020 [60]. This is currently under investigation by time series analysis.

The study design did not allow us to look for other enteric pathogens that cause diarrhoea, which could lead to both misattribution of index case illness to *Cryptosporidium*, and to overestimation of *Cryptosporidium* household transmission rates. We know that time constraints are a major contributor to issues in epidemiological observational studies, and in research are due in some part to ethical considerations [61, 62].

This work demonstrated that additional cases of *Cryptosporidium* occur in over a quarter of homes with a laboratory confirmed case. This is likely to affect up to a third of the household and cause considerable burden of illness. This is especially common where the index is a young child, with mums and other siblings are most at risk of secondary infection, and where homes have cases of *C. hominis*. This is important because current health care and public health systems are likely under ascertaining cases of sporadic illness, under examining person-to-person spread, and under-advising where specific clinical advice could be provided to high-risk households. Systematic changes that would provide improvement include species identification of all *Cryptosporidium* positive samples, fed routinely back into local health protection teams across England and Wales, and the consideration of specific clinical advice on prevention for high-risk households. This might include managing the patient’s expectations on the length of illness, and the possibility of relapse, and giving specific advice on preventing person-to-person spread [27].

Further work should expand on this research, which was only intended to be exploratory and low resolution. A better and closer examination of households and homes alongside a methodology to identify true secondary transmission more accurately should be the next step. This work should be designed in a way that allows correlations to be extrapolated more widely, and it is important that these are facilitated by all public health bodies across the UK.

## Supplementary Information

**Additional file 1:** Questionnaire data.

## Data Availability

The data that support the findings of this study are available from Professor Nigel Cunliffe at the University of Liverpool Institute of Infection, Veterinary, and Ecological Sciences. Restrictions apply to the availability of these data, which were used under license for the current study, and so are not publicly available. Data are however available from the authors upon reasonable request and with permission of Institute of Infection, Veterinary, and Ecological Sciences and the North West – Liverpool East NHS Research Ethics Committee.

## Abbreviations

BSS: Bristol stool scale
CI: Confidence interval
CRN: Clinical Research Network
CRU: Cryptosporidium Reference Unit
NHS: National Health Service
NIHR: National Institute for Health Research
OR: Odds ratio
PCR: Polymerase Chain Reaction
PHE: Public Health England
PHW: Public Health Wales
REC: Research Ethics Committee
UK: United Kingdom
