# Supplementary material for: Cross-sectional household transmission study of Cryptosporidium shows that C. hominis infections are a key risk factor for spread
Source: BMC Infect Dis. 2022 Feb 2;22:114. doi: 10.1186/s12879-022-07086-y (PMC8807379; doi:10.1186/s12879-022-07086-y)
Supplement: Supplementary file 1 — Additional file 1: Questionnaire data. [file 12879_2022_7086_MOESM1_ESM.pdf]

STUDY ID NUMBER:___________________________

The epiCrypt study

Investigating how *Cryptosporidium* infection spreads at home

Questionnaire & Consent Form

The epiCrypt Study_Ques & consent form V5.0 (25/06/2018)

Public Health England
UNIVERSITY OF LIVERPOOL | INSTITUTE OF INFECTION AND GLOBAL HEALTH
GIG CYMRU NHS WALES | Iechyd Cyhoeddus Cymru Public Health Wales

Who should complete this document?

It is best that the questionnaire is completed by the FIRST CASE, or their parent/guardian if the first case is under 16 years old.

The FIRST CASE is the person who had Crypto and was contacted by the research team, or their parent/guardian if they were a child.

This may not have been the first person in the home to be ill - don't worry. Still answer the questions about the FIRST CASE that was contacted by the research team.

- **BEFORE COMPLETING THIS QUESTIONNAIRE PLEASE READ ALL THE INFORMATION IN YOUR STUDY PACK.**
- **PLEASE INITIAL THE STATEMENTS ON THE CONSENT FORM ON THE FIRST PAGE OF THIS BOOKLET AND SIGN.**
- **WHEN YOU HAVE COMPLETED THE QUESTIONNAIRE, PLEASE RETURN IT IN THE FREEPOST ENVELOPE PROVIDED.**

CONSENT FORM

HOW TO COMPLETE THIS FORM

Each person who wants to take part should read the statements on the following page and initial in the box to show they understand them and agree.

Then add your names AND sign in the table.

Children aged 16 & 17 must consent for themselves.

Children under 16 years old may consent for themselves if a parent or guardian feels it is appropriate, or a parent or guardian may consent on their behalf. Just initial and sign this portion of the form to confirm.

You do not need to detach this form. Just return the whole questionnaire booklet. Returning these documents will be taken as consent to take part.

Each person in the household who wants to take part or is consenting for others must initial a box beside each statement

I have read, and understand, the information pack explaining the study and have been given the contact details of the study team if I have any questions					
I understand that taking part in this study is voluntary and I (or anyone I consent for) can leave at any time					
I understand that any stool sample I provide (or any I consent for) will be tested for Cryptosporidium species and genotypes					
I understand that all sample results are confidential, and my data may be shared securely with Public Health England/Public Health Wales to locate laboratory records relevant to this study					

We agree to take part in this study

NAME	SIGNATURE

IF YOU ARE ALSO CONSENTING FOR ANYONE UNDER 16 YEARS

Please mark with parent/guardian's initial

I am the parent/legal guardian of the child/children in the household and I consent to my child/children taking part in this study ☐ ☐ ☐

NAME OF PARENT/GUARDIAN	SIGNATURE	NAME OF CHILD

QUESTIONNAIRE

How to complete this questionnaire

Start at Section A on the following page

Write the date in the space at the top of the page

Some questions are about the first case and some are about the household. Follow the instructions to help.

You should:

- use pen to answer
- tick or cross your answers within the box like this: ☑
- follow any 'if YES' or 'if NO' instructions and leave blank any questions you do not need to answer

Don't worry if you don't want to answer anything – you can leave it blank. But it helps our research to get as much of the information as possible.

Some of the questions are about others in the household - these will be clearly worded. You may want to get some information from others in the home or some help answering some of the questions. This is fine.

Try to think about when you/the FIRST CASE had their Crypto illness.

You may have already answered some questions like this on a questionnaire, or for your local environmental health officer, but we need to get these answers separately on this questionnaire.

Just answer as best you can – any information helps.

Remember: The FIRST CASE is the person who had Crypto and was contacted by the research team, or their parent/guardian if they were a child.

This may not have been the first person in the home to be ill - don't worry. Still answer the questions about the FIRST CASE that was contacted by the research team.

DATE FILLED IN ______/______/______

SECTION A

A1. What is the postcode of the home? ________________________

A2. How many people live in the household in total (including the first case)?

____

Please fill in the table below telling us how many people live in the household in each of the age groups

Aged less than 1 year	Aged 1-3 years	Aged 4-5 years	Aged 6–14 years	Aged 15-24 years	Aged 25-34 years	Aged 35–44 years	Aged 45-64 years	Aged 65 or over

A3. How old is the FIRST CASE?

Date of Birth ☐☐ / ☐☐ / ☐☐☐☐ OR Age __________

A4. What symptoms did the FIRST CASE have when they had Crypto?

(Tick all that apply)

☐ Diarrhoea (3 or more loose stools/runny poos in a day)

☐ Feeling sick/nausea ☐ Vomiting/being sick

☐ Stomach pain/Cramps ☐ High temperature/feverish ☐ Headache

☐ Other ____

____

A5. When did the FIRST CASE start to feel ill with these symptoms?

__ __ / __ __ / __ __ __ __

A6. Is the FIRST CASE still ill with these symptoms?

- ☐ Yes
- ☐ No

If YES, and they are still unwell, for how many days have they had it now?

____

If NO, and they are better now, for how many days did the symptoms last?

____

A7. In the TWO WEEKS before symptoms started, did the FIRST CASE have close contact with anyone else who had diarrhoea and/or vomiting?

- ☐ Yes
- ☐ No

If YES, was that someone who lives:

- ☐ In the home?
- ☐ Elsewhere?

A8. In the TWO WEEKS before or after the FIRST CASE was ill, has anyone ELSE in the home been ill with the same kind of symptoms?

- ☐ Yes
- ☐ No

Who's who in the household and who else has been ill?

Please fill in **table A** on the next page, including all members of the household that are taking part in the study. This will be all the people on the consent form on the front of this booklet.

Please tell us whether others in the household have also been ill with diarrhoea or vomiting recently or around the same time as the FIRST CASE.

Try to think roughly about the month before and after the FIRST CASE was ill.

Don't worry if the dates are estimates – as accurate as you can get is fine. If you cannot remember, just let us know if you think it was before or after the first case started to get ill. This is still useful information.

Adding the age and sex helps us to identify the household members and match up their samples.

Look at the example to help:

Age	Sex	Relationship to first case	Been ill with diarrhoea and/or vomiting (Yes/No/ Don't know)	When they became ill (Date if known, otherwise before/ after the first case)	How many days were they ill with these symptoms?	Did they see a doctor about this illness?
39	*F*	*Mother*	*Yes*	*18/12/2017*	*10*	*No*
42	*M*	*Father*	*Yes*	*Before*	*About 3 days*	*No*
6	*M*	*Brother*	*No*	-	-	-
24	*F*	*Lodger/ Housemate*	*Don't know*	-	-	-

TABLE A.

Age	Sex	Relationship to first case	Been ill with diarrhoea and/or vomiting (Yes/No/ Don't know)	When they became ill (Date if known, otherwise before/ after the first case)	How many days were they ill with these symptoms?	Did they see a doctor about this illness?

SECTION B

B1. Sex of FIRST CASE

- ☐ Female
- ☐ Male
- ☐ Other
- ☐ Prefer not to say

B2. Is the FIRST CASE:

(Tick all that apply)

- ☐ At nursery or pre-school
- ☐ In education (school/college/uni etc.)
- ☐ Working (paid work, either outside the home or from home)
- ☐ A stay at home parent or carer
- ☐ Retired
- ☐ Other

If OTHER, please describe

____

If the FIRST CASE is working, what is their main occupation?

____

____

B3. Does the FIRST CASE have any long-term medical problems or long-term illnesses?

- ☐ Yes
- ☐ No

If YES, please describe

____

Activities of the first case and others in the home in the 2 weeks before illness

Please fill in table B on the next page.

We want to find out if the household has been involved in any activities in the table.

Please fill in the first column in the table with a cross or tick if the FIRST CASE did any of these things in the **TWO WEEKS** before their symptoms started.

Also tick the second column if anyone else who lives in the home was there and did those things at the same time.

Don't worry if you're not too sure – as much as you can remember is fine. This is still useful information.

Look at the short example to help:

ACTIVITY	***FIRST CASE***	***ANYONE ELSE IN THE HOME***
Travel outside the UK	✔	✔
Swimming – outdoors (lake, river, stream etc.)		
Swimming – in a treated swimming pool, either indoors or outdoors (such as	✔	

TABLE B.

ACTIVITY	FIRST CASE	ANYONE ELSE IN THE HOME
Travel outside the UK		
Swimming – outdoors (lake, river, stream etc.)		
Swimming – in a treated swimming pool, either indoors or outdoors (such as a pool at a leisure centre or a lido)		
Other water activities/sport (such as surfing, rowing, water-skiing etc.)		
Other outdoor activities (such as camping, climbing, hiking, cycling etc.)		
Gardening (at home or elsewhere, such as an allotment)		
Contact with pets (at home or with pets at another house)		
Visiting or working on a farm or had contact with farm animals		
Visiting or working at a zoo or had contact with zoo or wild animals		

SECTION C

C1. How many bedrooms are there in the house? ________________

Do any adults share a bedroom to sleep in at night?

☐ Yes

☐ No

Do any children (under 18 years old) share a bedroom to sleep in at night?

☐ Yes

☐ No

Do any infants (under 1 year) sleep in a bedroom with an adult at night?

☐ Yes

☐ No

C2. Does the FIRST CASE regularly (more than once a week) share a bed with anyone else in the home?

☐ Yes

☐ No

C3. How many bathrooms are there? __________________

How many other people use the same bathroom at home as the FIRST CASE?

1	2	3	4	5	6	7	8	9	10
☐	☐	☐	☐	☐	☐	☐	☐	☐	☐

C4. How many toilets/WCs are there in total? ________________

How many other people use the same toilet at home as the FIRST CASE?

1	2	3	4	5	6	7	8	9	10
☐	☐	☐	☐	☐	☐	☐	☐	☐	☐

C5. Does the FIRST CASE regularly (more than once a week) share a bath with anyone else in the home?

☐ Yes

☐ No

C6. Is your home on, or part of, a farm or smallholding?

☐ Yes

☐ No

C7. Does your home have any outside space/area?

☐ Yes

☐ No

If YES, what kind? *(Tick all that apply)*

☐ Yard (no grass)

☐ Front garden

☐ Back garden

☐ Other ____

C8. Is your home supplied by mains water (i.e. a water company that you get a bill from)?

☐ Yes

☐ No

C9. Are there any pets in the home?

☐ Yes

☐ No

If YES, what kind? *(Tick all that apply)*

☐ Cat(s) ☐ Bird(s ☐ Reptile(s)

☐ Dog(s) ☐ Horse(s) ☐ Fish ☐ Other ______________

C10. Are there any other animals at or around the home - such as livestock, chickens, cows, sheep etc.?

☐ Yes

☐ No

If YES, please describe

____

____

SECTION D

D1. Is the FIRST CASE a child under 5 years old?

- ☐ Yes
- ☐ No

If YES, are they? *(Tick all that apply)*

- ☐ In nappies
- ☐ Potty/Toilet training
- ☐ Neither

If NO, are they an older child (more than 5 years old) or an adult that needs help with any of the following? *(Tick all that apply)*

- ☐ Nappy changing
- ☐ Assisting with going to the toilet
- ☐ Bathing
- ☐ None of the above

D2. Does the FIRST CASE regularly (more than once a week) help a child (or children) under 5 with any of the following? (this could be at work or in the home)

(Tick all that apply)

- ☐ Nappy changing
- ☐ Potty training
- ☐ Assisting with the toilet
- ☐ Bathing
- ☐ None of the above/not applicable

D3. Does the FIRST CASE regularly (more than once a week) help a child over 5, or an adult, with any of the following? (this could be at work or in the home)

(Tick all that apply)

- ☐ Nappy changing
- ☐ Potty training
- ☐ Assisting with the toilet
- ☐ Bathing
- ☐ None of the above/not applicable

D4. Are there ANY children (under 5) living in the household who are in nappies and/or currently toilet training?

- ☐ Yes
- ☐ No

If YES, when changing nappies do you/others in the home generally use any of the following: *(Tick all that apply)*

Don't worry if it applies to more than one child – just tick if anyone in the home generally uses these things when changing nappies or helping the children with the toilet

- ☐ Disposable nappies
- ☐ Cloth nappies
- ☐ Disposable baby wipes/wet wipes
- ☐ Water and cotton wool
- ☐ Top/Tail bowl
- ☐ Nappy cream
- ☐ Hand gel
- ☐ Nappy bags
- ☐ A nappy bin
- ☐ A changing mat

D5. Does the FIRST CASE regularly/usually prepare meals or cook for others in the household?

- ☐ Yes
- ☐ No

If YES, did they still prepare meals or cook for the rest of the household while they were ill with Crypto?

- ☐ Yes
- ☐ No
- ☐ Don't know

D6. When hand-washing, do household members normally use:

(Tick all that apply)

- ☐ Hot water
- ☐ Soap (bar or hand wash)
- ☐ Antibacterial hand wash
- ☐ Alcohol gel
- ☐ Hand wipes
- ☐ Don't know
- ☐ None of the above/Not applicable

D7. Generally, do household members wash their hands before and/or after any of the following?

If you're not sure, just try to think about what most people in the home do

Going to the toilet

- ☐ Before
- ☐ After
- ☐ Both
- ☐ Neither

Helping someone with the toilet

(if applicable)

- ☐ Before
- ☐ After
- ☐ Both
- ☐ Neither

Nappy changing (if applicable)

- [ ] Before
- [ ] After
- [ ] Both
- [ ] Neither

Eating

- [ ] Before
- [ ] After
- [ ] Both
- [ ] Neither

Preparing food

- [ ] Before
- [ ] After
- [ ] Both
- [ ] Neither

Handling dirty/soiled laundry

- [ ] Before
- [ ] After
- [ ] Both
- [ ] Neither

THANK YOU FOR COMPLETING THE QUESTIONNAIRE!

Please let us know if you would like a summary of the results when we have finished our study.

- [ ] Yes
- [ ] No

Please let us know if you would be willing to be contacted again if we had any further questions about your illness or your household.

- [ ] Yes
- [ ] No

If YES to either of the above, please give the preferred contact details:

- [ ] Email ____________________
- [ ] Post ____________________
- [ ] Telephone ____________________

Please place in the FREEPOST ENVELOPE PROVIDED and return to the epiCrypt study team. Thank you!
